# The impact of clopidogrel combined with aspirin or aspirin alone on prognosis and adverse events in patients undergoing coronary artery bypass grafting in the first month after surgery

**DOI:** 10.3389/fcvm.2025.1600353

**Published:** 2025-06-23

**Authors:** Haiming Li, Hui Hu, Jingxing Li, Chengxiong Gu, Bo Li

**Affiliations:** Department of Cardiovascular Surgery, Beijing Anzhen Hospital, Capital Medical University, Beijing, China

**Keywords:** coronary artery bypass grafting, dual antiplatelet therapy, aspirin, clopidogrel, graft occlusion, postoperative outcomes

## Abstract

**Background:**

Coronary artery bypass grafting (CABG) was a critical intervention for patients with obstructive coronary artery disease, yet managing thrombotic complications post-surgery remains challenging. Aspirin was the standard antiplatelet therapy following CABG; however, the potential benefits of dual antiplatelet therapy (DAPT) with clopidogrel were not well-defined in this setting. This study evaluates the impact of DAPT compared to aspirin alone on prognosis and adverse events in the first month after CABG.

**Methods:**

This retrospective cohort study analyzed data from 281 patients undergoing CABG from August 2020 to July 2023. Using propensity score matching (PSM), these patients were separated into two groups based on their postoperative medication: the aspirin alone group (ASA, *n* = 117) and the dual antiplatelet therapy group (DAPT, *n* = 117). PSM ensured balanced baseline characteristics. Graft patency, platelet function, and postoperative adverse events were evaluated, with statistical significance set at a *P*-value < 0.05. Categorical variables were reported as frequencies and percentages [*n* (%)] and analyzed using Chi-square test. Continuous variables with a normal distribution were presented as mean ± standard deviation (SD) and analyzed using *t* test.

**Results:**

DAPT resulted in significantly lower rates of saphenous vein graft occlusion compared to aspirin alone (17.09% vs. 29.06%, *P* = 0.030). Inhibition of platelet aggregation (IPA) was enhanced in the DAPT group (64.35% vs. 62.46%, *P* = 0.010), and thromboxane B2 levels were consistently lower on days 7, 14, and 30 post-surgery (*P* = 0.009, *P* = 0.005, and *P* = 0.002, respectively). Although adverse cardiovascular events did not significantly differ, minor bleeding, such as epistaxis, was more common in the DAPT group (6.84% vs. 0.85%, *P* = 0.041). Regression analysis showed DAPT reduced the odds of adverse events (OR: 0.452, 95% CI: 0.253–0.816, *P* = 0.008).

**Conclusion:**

DAPT with clopidogrel and aspirin improves graft patency and platelet inhibition in the first month following CABG, though it was associated with increased minor bleeding events.

## 1 Introduction

Coronary artery bypass grafting (CABG) remains a cornerstone treatment for patients with obstructive coronary artery disease (CAD), particularly in cases where medical management and percutaneous coronary interventions (PCI) were less effective (1). Despite advancements in surgical techniques and postoperative care, managing patients in the immediate postoperative period following CABG was critical to improving outcomes and minimizing adverse events (2). Antiplatelet therapy, primarily with aspirin, was well-established as a standard intervention to reduce thrombotic complications (3). Nonetheless, the optimal antiplatelet strategy in the perioperative period remains controversial, with growing interest in the potential benefits of dual antiplatelet therapy (DAPT) (4).

Clopidogrel, a P2Y12 receptor inhibitor, in combination with aspirin, has been extensively studied in patients undergoing PCI, demonstrating significant reductions in thrombotic events (5). However, its role in the CABG setting, particularly in the first month post-surgery when the risk of graft occlusion was highest, was not as well defined (6, 7). The American College of Cardiology/American Heart Association (ACC/AHA) guidelines have provided recommendations for DAPT use post-CABG, yet these guidelines were primarily extrapolations from PCI data due to the paucity of CABG-specific robust clinical trials (8). Therefore, a critical evaluation of clopidogrel in combination with aspirin compared to aspirin monotherapy was warranted to elucidate the impact on patient outcomes and adverse events (9).

Aspirin monotherapy has traditionally been the mainstay antiplatelet regimen post-CABG (10). It inhibits platelet aggregation by irreversibly inhibiting cyclooxygenase−1 (COX-1), thereby reducing thromboxane A₂ production (10). Aspirin's effectiveness in secondary prevention of cardiovascular events, including in patients following CABG, was well-established, reducing the risk of graft thrombosis and subsequent ischemic events (11, 12). However, aspirin alone may not be sufficient in all patients, particularly those with high thrombotic risk or inadequate platelet inhibition, which invites consideration of additional therapies such as clopidogrel (13).

Clopidogrel, through its active metabolite, selectively inhibits the binding of ADP to its platelet receptor, thereby impeding ADP-mediated activation of the glycoprotein GPIIb/IIIa complex and subsequent platelet aggregation (14). The pharmacodynamic synergy between clopidogrel and aspirin has been consistently demonstrated in the context of acute coronary syndrome and PCI, improving cardiovascular outcomes (14). Nonetheless, the extension of these benefits to CABG patients, balanced against potential hemorrhagic risks associated with dual therapy, requires further exploration. This study aims to systematically assess the impact of clopidogrel combined with aspirin against aspirin alone on the prognosis and adverse events in patients during the critical first month following CABG surgery.

## 2 Materials and methods

### 2.1 Ethics statement

This research received approval from our institution's Institutional Review Board and Ethics Committee (No.Chictr2400085741). Due to the study's retrospective nature and sole use of de-identified patient data, which presents no risk or impact on patient care, informed consent requirements were waived. This waiver aligns with regulatory and ethical standards for retrospective research and was duly authorized by our Institutional Review Board and Ethics Committee.

### 2.2 Study design

This retrospective cohort study analyzed the medical records of 281 patients who underwent CABG at our hospital between August 2020 and July 2023. Patient data were extracted from the medical record system. Based on the postoperative medications, patients were categorized into two groups: the aspirin alone group (ASA) and the aspirin plus clopidogrel group (DAPT).

### 2.3 Administration of antiplatelet drugs

Patients who underwent successful CABG were categorized into two groups: the ASA group, which received 100 mg of aspirin daily (Bufferin 100, Takeda Pharmaceutical, Japan), and the DAPT group, which received a combination of 100 mg of aspirin and 75 mg of clopidogrel daily (Plavix, Bristol-Myers Squibb, United States). Oral antiplatelet therapy commenced once clinical stability was confirmed and when chest tube output decreased to 30 ml/h. All patients began antiplatelet medication within 24 h post-surgery and continued this regimen for one month thereafter.

### 2.4 Study population

Inclusion Criteria: Participants were required to have multivessel CAD as defined by the American Heart Association (AHA) and be unsuitable candidates for PCI (15). Additional criteria included being over 18 years of age, undergoing primary isolated CABG with or without cardiopulmonary bypass, and having complete medical records. For those in the DAPT group, carrying the CYP2C19 genotype with two normally functioning alleles was also necessary.

Exclusion Criteria: Patients were excluded if they had experienced a myocardial infarction (MI) within the preceding 48 h, presented with cardiogenic shock, had a history of atrial fibrillation, or were receiving ongoing oral anticoagulation therapy. Other exclusion factors included a stroke within the last 30 days, active bleeding, known coagulopathy, liver disease, a history of peptic ulcers, contrast media allergy, mandatory clopidogrel treatment (such as due to a stent or previous stroke), an allergy to study medications, or substance abuse issues such as alcohol or narcotics. Pregnant individuals were also excluded.

### 2.5 Data collection and processing

A total of 281 patient records were initially selected for analysis, with 47 patients excluded after propensity score matching (PSM). The resulting retrospective cohort comprised 117 patients in the aspirin (ASA) group and 117 in the DAPT group. Tables 1, 2 provide the baseline characteristics of both groups before and after 1:1 PSM. Post-matching, the baseline characteristics were well-balanced, with standardized mean differences (SMDs) consistently below 0.1. Initially, the ASA group exhibited significantly lower body mass index (BMI) values and platelet counts compared to the DAPT group, along with a lower proportion of patients with hypertension. However, following PSM, no significant differences were observed between the two groups in terms of gender distribution, mean age, and average BMI.

**Table 1:** Demographic characteristics before PSM.

Parameters	ASA group (*n* = 125)	DAPT group (*n* = 156)	t/*χ*^2^	*P*
Male	98 (78.40%)	123 (78.85%)	0.008	0.928
Age	59.71 ± 8.87	61.15 ± 8.79	1.356	0.176
BMI	22.52 ± 2.61	23.25 ± 2.78	2.272	0.024
Current smoker	55 (44.00%)	73 (46.79%)	0.219	0.640
Drinker	32 (25.60%)	47 (30.13%)	0.704	0.401
Hypertension	101 (80.80%)	139 (89.10%)	3.839	0.050
CHD duration (months)	15.01 ± 7.89	14.03 ± 6.21	1.137	0.257
Vessel disease			0.102	0.950
One-vessel disease	23 (18.40%)	31 (19.87%)		
Two-vessel disease	64 (51.20%)	79 (50.64%)		
Three-vessel disease	38 (30.40%)	46 (29.49%)		
History of therapy				
Aspirin	99 (79.20%)	124 (79.49%)	0.003	0.953
Clopidogrel	41 (32.80%)	50 (32.05%)	0.018	0.894
Ticagrelor	18 (14.40%)	25 (16.02%)	0.141	0.707
ADP-receptor inhibitor	56 (44.80%)	69 (44.23%)	0.009	0.924
Statin	109 (87.20%)	134 (85.90%)	0.101	0.751
Angina CCS class			0.156	0.693
I or II	46 (36.80%)	61 (39.10%)		
III or IV	80 (64.00%)	95 (60.90%)		
Euroscore	4.71 ± 2.63	4.89 ± 2.71	0.584	0.560
CHD family history	19 (15.20%)	26 (16.67%)	0.111	0.739
On-pump	79 (63.20%)	86 (55.13%)	1.865	0.172
Off-pump	46 (36.80%)	70 (44.87%)		
LVEF (%)	47.97 ± 8.31	46.52 ± 8.53	1.445	0.153
Creatinine, μmol/L	89.03 ± 18.36	86.48 ± 16.78	1.211	0.224
Platelet count, ×10^9^/L	278.02 ± 96.71	303.65 ± 100.80	2.157	0.032
Fibrinogen, g/L	14.60 ± 2.93	14.59 ± 3.81	0.023	0.982
aPTT, s	29.43 ± 4.15	29.14 ± 6.38	0.470	0.639

BMI, body mass index; CHD, coronary heart disease; ADP, adenosine diphosphate; CCS, Canadian cardiovascular society; LVEF, left ventricular ejection fraction; aPTT, activated partial thromboplastin time.

**Table 2:** Demographic characteristics after PSM.

Parameters	ASA group (*n* = 117)	DAPT group (*n* = 117)	t/χ^2^	*P*
Male	93 (79.49%)	94 (80.34%)	0.027	0.870
Age	59.87 ± 8.73	60.09 ± 8.65	0.196	0.845
BMI	22.82 ± 2.66	22.97 ± 2.37	0.456	0.649
Current smoker	51 (43.59%)	54 (46.15%)	0.155	0.693
Drinker	30 (25.64%)	33 (28.20%)	0.195	0.658
Hypertension	98 (83.76%)	107 (91.45%)	3.188	0.074
CHD duration (months)	16.02 ± 7.53	15.03 ± 7.33	1.020	0.309
Vessel disease			0.022	0.989
One-vessel disease	21 (17.95%)	21 (17.95%)		
Two-vessel disease	60 (51.28%)	61 (52.14%)		
Three-vessel disease	36 (30.77%)	35 (29.91%)		
History of therapy				
Aspirin	96 (82.05%)	97 (82.90%)	0.030	0.863
Clopidogrel	38 (32.48%)	37 (31.62%)	0.019	0.889
Ticagrelor	16 (13.68%)	18 (15.38%)	0.138	0.711
ADP-receptor inhibitor	55 (47.01%)	53 (45.30%)	0.069	0.793
Statin	106 (90.60%)	105 (89.74%)	0.048	0.826
Angina CCS class			0.019	0.891
I or II	41 (35.04%)	42 (35.90%)		
III or IV	76 (64.96%)	75 (64.10%)		
Euroscore	4.73 ± 2.55	4.82 ± 2.74	0.275	0.783
CHD family history	17 (14.53%)	16 (13.68%)	0.035	0.851
On-pump	77 (65.81%)	71 (60.68%)	0.662	0.416
Off-pump	40 (34.19%)	46 (39.32%)		
LVEF (%)	46.78 ± 8.25	46.65 ± 8.29	0.121	0.904
Creatinine, μmol/L	88.55 ± 17.91	87.70 ± 16.25	0.378	0.706
Platelet count, × 10^9/L	297.13 ± 92.95	298.04 ± 95.61	0.074	0.941
Fibrinogen, g/L	14.63 ± 2.84	14.52 ± 3.44	0.264	0.792
aPTT, s	29.34 ± 3.98	29.21 ± 5.58	0.198	0.843

Among the 234 patients who underwent surgery, 50% (117 patients) received DAPT, and 50% (117 patients) received aspirin alone within the first month post-surgery. Baseline demographic and clinical data, including age, gender, and BMI, were obtained from the medical record system. During the one-year follow-up period, events were retrospectively assessed, medication adherence was verified, and blood samples, MSCT scans, and other examinations were conducted.

The Canadian Cardiovascular Society Grading Scale for Angina Pectoris was outlined as follows:

Class I: Ordinary physical activity does not induce angina. Angina only occurs with strenuous, rapid, or prolonged exertion during work or recreational activities.
Class II: There was a slight limitation of ordinary activities. Angina occurs with rapid walking or stair climbing, walking uphill, or walking or stair climbing after meals, in cold or windy conditions, under emotional stress, or within the first few hours after awakening. Angina may also occur when walking more than two blocks on level ground or climbing more than one flight of stairs at a normal pace under normal conditions.
Class III: There was a marked limitation of ordinary physical activity. Angina occurs after walking one or two blocks on level ground or climbing a single flight of stairs at a normal pace and in normal conditions.
Class IV: There was an inability to engage in any physical activity without discomfort, as angina may be present even at rest (16).

Multislice computed tomography (MSCT) was utilized to evaluate graft patency in patients following CABG. All scans were conducted with a 320-slice scanner (Aquilion ONE, Toshiba, Tokyo, Japan). A cardiac MSCT specialist analyzed the images using the Vitrea software package (Vital Images, USA).

Venous blood samples were collected from patients at 1, 7, 14, and 30 days post-surgery. Plasma was isolated via centrifugation for analysis. ELISA kits (Human Thromboxane B2 ELISA Kit, Cayman Chemical, USA) were employed, with the TXB2 antigen (CEA877Ge, Cloud-Clone Corp, USA) added to the microplate wells. Serum samples and various standard concentrations were introduced into the wells to facilitate antigen-antibody reactions. Following a wash phase, enzyme-labeled detection reagents were added. Substrate solution was subsequently introduced, reacting with the enzyme to induce a color change. Once the desired intensity was reached, a stop solution was applied to halt the reaction. Absorbance at a specific wavelength was measured using an ELISA reader (SpectraMax i3x, Molecular Devices, USA). A standard curve, based on the absorbance of the standards, was used to determine TXB2 concentrations in the samples. The levels of TXB2 serve as indicators of platelet activity and coagulation status, which aid in predicting cardiovascular events and assessing the effectiveness of surgical interventions.

On the seventh day post-surgery, whole blood samples were collected from patients and placed into cuvettes for analysis using a light transmission aggregometer (Model 490-2D, Chrono-Log Corporation, USA). The instrument measured and recorded the baseline light transmission through the samples. ADP (P/N 384, Chrono-Log Corporation, USA) was added to initiate platelet aggregation, and the aggregometer continuously monitored and recorded changes in light transmission over time. The analysis software then converted raw light intensity data into aggregation rates to quantify the extent and speed of platelet aggregation. Additionally, samples treated with ADP were placed in specialized electrode cups where resistance changes were measured by the MEA device (Maestro MEA, Axion Biosystems, USA), which quantifies platelet aggregation by detecting these resistance changes.

Following surgery, a 3D ultrasound machine (Siemens Healthineers ACUSON SC2000 PRIME, Siemens Healthineers, Germany) was employed to construct a comprehensive 3D model of the heart from a series of 2D images captured from various planes. The built-in software analysis tools calculated the left ventricular ejection fraction (LVEF) using these high-quality 3D images. By comparing the calculated LVEF values to the normal range (50%–70%), the extent of LVEF decline in patients was assessed. A significant decrease in LVEF values suggests a potentially poorer long-term survival and quality of life, making it a crucial parameter for evaluating patient prognosis.

An LVEF of 50% or greater was considered normal, while a range of 40% to 50% indicates a mild reduction. An LVEF between 30% and 40% suggests a moderate reduction, and an LVEF below 30% signifies a severe reduction.

During the 30-day postoperative follow-up, venous blood samples were collected from the patients for analysis. These samples were processed according to experimental protocols and measured using an optical aggregometer (Chrono-Log Corporation, USA), a device based on light scattering principles to detect platelet aggregation. The processed blood samples were placed in test tubes, and Adenosine Diphosphate (Chrono-Log Corporation, USA) was used to induce platelet aggregation. Changes in light transmission through the samples were detected and converted into electrical signals by the aggregometer. These signals were recorded to evaluate the extent of platelet aggregation, which indicated the degree of inhibition by the drug.

Simultaneously, the blood samples were centrifuged to separate the plasma. The platelet-rich and platelet-poor plasma were then exposed to Adenosine Diphosphate to stimulate aggregation, and were analyzed using a platelet analyzer (Model 700 Lumi-aggregometer, Chrono-Log Corporation, USA) via light transmittance aggregometry. This system calculated Platelet Reactivity Index (PRI) values. The results for Inhibition of Platelet Aggregation (IPA) and PRI provided insights into platelet function, which were critical in improving patient prognosis.

Adverse events such as reoperation within one year post-surgery, various types of postoperative bleeding, and MI were documented from the medical record system and subjected to statistical analysis.

### 2.6 Statistical analysis

The PSM was conducted using the MatchIt package in R (version 4.3.2). We evaluated the balance of baseline covariates between groups both before and after PSM using the SMD, where an SMD greater than 0.1 indicates covariate imbalance.

Data analysis was performed using SPSS statistical software version 29.0 (IBM Corp., Armonk, NY, USA). Categorical variables were reported as frequencies and percentages [*n* (%)] and analyzed using Chi-square test. Continuous variables with a normal distribution were presented as mean ± standard deviation (SD) and analyzed using *t* test. Statistical significance was determined by a *p*-value of less than 0.05 for all tests.

## 3 Results

### 3.1 Demographic and basic data

Prior to PSM, the demographic characteristics of the ASA (aspirin alone) group (*n* = 125) and the DAPT group (*n* = 156) were compared as shown in Table 1. The distribution of male participants was nearly identical between groups, with 78.40% in the ASA group and 78.85% in the DAPT group (*P* = 0.928). Mean age was slightly higher in the DAPT group (61.15 ± 8.79 years) compared to the ASA group (59.71 ± 8.87 years), though this was not statistically significant (*P* = 0.176). A significant difference was observed in BMI between the groups (ASA: 22.52 ± 2.61; DAPT: 23.25 ± 2.78; *P* = 0.024). There were no significant differences in current smoker status, drinking habits, or coronary heart disease (CHD) duration. The prevalence of hypertension was notably higher in the DAPT group (89.10%) compared to the ASA group (80.80%), with a borderline significance (*P* = 0.050). The distribution of vessel disease, history of therapy, angina severity, and various laboratory parameters did not show significant differences. Although platelet count was higher in the DAPT group (303.65 ± 100.80 × 10^9^/L) than in the ASA group (278.02 ± 96.71 × 10^9^/L), the difference reached statistical significance (*P* = 0.032). Other parameters such as LVEF, creatinine levels, fibrinogen, and activated partial thromboplastin time (aPTT) were comparable between the two groups, indicating a generally well-matched patient population apart from the noted variables. These results suggest no substantial demographic or baseline clinical differences that could potentially confound the comparative analysis of postoperative outcomes between the two treatment strategies.

After PSM, the demographic and baseline characteristics of patients in the ASA group (*n* = 117) and the DAPT group (*n* = 117) demonstrated well-balanced groups with no statistically significant differences between them, as presented in Table 2. The proportion of male participants was similar in both groups, with 79.49% in the ASA group and 80.34% in the DAPT group (*P* = 0.870). The average age of patients was comparable, with 59.87 ± 8.73 years in the ASA group and 60.09 ± 8.65 years in the DAPT group (*P* = 0.845). BMI, smoking status, and alcohol consumption were similar across groups, with BMIs of 22.82 ± 2.66 in the ASA group and 22.97 ± 2.37 in the DAPT group (*P* = 0.649). The incidence of hypertension was slightly higher in the DAPT group (91.45%) than in the ASA group (83.76%), although this did not reach statistical significance (*P* = 0.074). Other characteristics, including CHD duration, vessel disease status, history of therapy, angina Canadian Cardiovascular Society (CCS) class, and Euroscore, were evenly distributed between the two groups. The laboratory indices such as LVEF, creatinine levels, platelet count, fibrinogen, and aPTT also showed no significant differences. The balanced distribution of these variables following PSM suggests that the comparisons of outcomes between the ASA and DAPT groups was less likely confounded by baseline characteristics, which allows for more reliable assessment of the treatment effects on postoperative prognosis and adverse events in these CABG patients.

The prevalence of prior stroke was identical in both groups, with only 1 case (0.85%) each (*P* = 1.000) (Table 3). The incidence of diabetes mellitus (DM) was slightly higher in the DAPT group at 21.37% compared to 19.66% in the ASA group, but this difference was not statistically significant (*P* = 0.746). The frequency of hyperlipidemia was comparable between the ASA group (39.32%) and the DAPT group (36.75%) (*P* = 0.686). Chronic obstructive pulmonary disease (COPD) and peripheral vascular disease (PVD) were present in a small proportion of patients, with similar occurrence in both groups (COPD: 5.98% ASA vs. 7.69% DAPT, *P* = 0.604; PVD: 7.69% ASA vs. 5.98% DAPT, *P* = 0.604). While the percentage of patients experiencing MI within the past year showed a trend towards being lower in the DAPT group (11.11%) compared to the ASA group (19.66%), this did not reach statistical significance (*P* = 0.070). Other factors such as PCI within the year, unstable angina (UA), use of saphenous vein grafts (SVG), presence of hypercholesterolemia (Hyperchol), left main disease (LMD), and previous heart surgery showed similar distributions between the two groups. These findings indicate consistency in medical histories across both treatment groups, suggesting a low likelihood of confounding effects from these baseline health conditions when analyzing the impact of the treatment strategies on patient outcomes post-CABG.

**Table 3:** Medical history investigation.

Parameters	ASA group (*n* = 117)	DAPT group (*n* = 117)	χ^2^	*P*
Prior stroke	1 (0.85%)	1 (0.85%)	0.000	1.000
DM	23 (19.66%)	25 (21.37%)	0.105	0.746
Hyperlipidemia	46 (39.32%)	43 (36.75%)	0.163	0.686
COPD	7 (5.98%)	9 (7.69%)	0.268	0.604
PVD	9 (7.69%)	7 (5.98%)	0.268	0.604
MI within 1 year	23 (19.66%)	13 (11.11%)	3.283	0.070
PCI within 1 year	8 (6.84%)	13 (11.11%)	1.308	0.253
UA	36 (30.77%)	44 (37.61%)	1.216	0.270
SVG	83 (70.94%)	90 (76.92%)	1.087	0.297
Hyperchol	111 (94.87%)	113 (96.58%)	0.418	0.518
LMD	20 (17.09%)	23 (19.66%)	0.256	0.613
Previous heart surgery	1 (0.85%)	1 (0.85%)	0.000	1.000

DM, diabetes mellitus; COPD, chronic obstructive pulmonary disease; PVD, peripheral vascular disease; MI, myocardial infarction; PCI, percutaneous coronary intervention; UA, unstable angina; SVG, saphenous vein graft; LMD, left main disease.

### 3.2 Graft characteristics

The distribution of the number of grafts per patient was similar between the groups, with 7.69% of ASA patients and 6.83% of DAPT patients receiving two grafts, 76.92% and 77.78% receiving three grafts, and 15.38% in both groups receiving four grafts (*P* = 0.968) (Table 4). The average number of grafts per patient was nearly identical, with the ASA group having a mean of 3.04 grafts (356 total grafts for 117 patients) and the DAPT group averaging 3.08 grafts per patient (361 total grafts for 117 patients). This consistent graft distribution across both treatment groups indicates that any differences in postoperative outcomes were unlikely to be influenced by variations in graft characteristics, allowing a more direct assessment of the therapeutic effects of clopidogrel with aspirin compared to aspirin alone in the recovery trajectory post- CABG within the critical first month.

**Table 4:** Graft characteristics of patients.

Parameters	ASA group (*n* = 117)	DAPT group (*n* = 117)	χ^2^	*P*
Number of grafts			0.064	0.968
2	9 (7.69%)	8 (6.83%)		
3	90 (76.92%)	91 (77.78%)		
4	18 (15.38%)	18 (15.38%)		
Grafts/patients	3.04 (356/117)	3.08 (361/117)		

LAD, left anterior descending coronary artery; LCx, left circumflex coronary artery; RCA, right coronary artery; LIMA, left internal mammary artery; RA, radial artery; SVG, saphenous vein graft.

### 3.3 Prognostic condition

The overall incidence of graft occlusion was higher in the ASA group, with 36 patients (30.77%) experiencing occlusion compared to 23 patients (19.66%) in the DAPT group, a difference approaching statistical significance (*P* = 0.050) (Table 5). Specifically, SVG occlusions were significantly more frequent in the ASA group, affecting 34 patients (29.06%) vs. 20 patients (17.09%) in the DAPT group (*P* = 0.030). In contrast, the rate of left internal mammary artery (LIMA) and radial artery (RA) occlusions was low and did not differ significantly between groups, with LIMA occlusions in 1 ASA patient (0.85%) and 2 DAPT patients (1.71%), and RA occlusion occurring in 1 patient (0.85%) in each group (*P* = 1.000 for both LIMA and RA). These findings indicate that the use of clopidogrel in combination with aspirin may reduce the risk of SVG occlusion relative to aspirin alone, potentially contributing to improved short-term graft patency in patients undergoing CABG.

**Table 5:** Prevalence of graft occlusion.

Type	ASA group (*n* = 117)	DAPT group (*n* = 117)	χ^2^	*P*
LIMA	1 (0.85%)	2 (1.71%)	0.000	1.000
RA	1 (0.85%)	1 (0.85%)	0.000	1.000
SVG	34 (29.06%)	20 (17.09%)	4.719	0.030
Total	36 (30.77%)	23 (19.66%)	3.830	0.050

LIMA, left internal mammary artery; RA, radial artery; SVG, saphenous vein graft.

**Figure 1:**
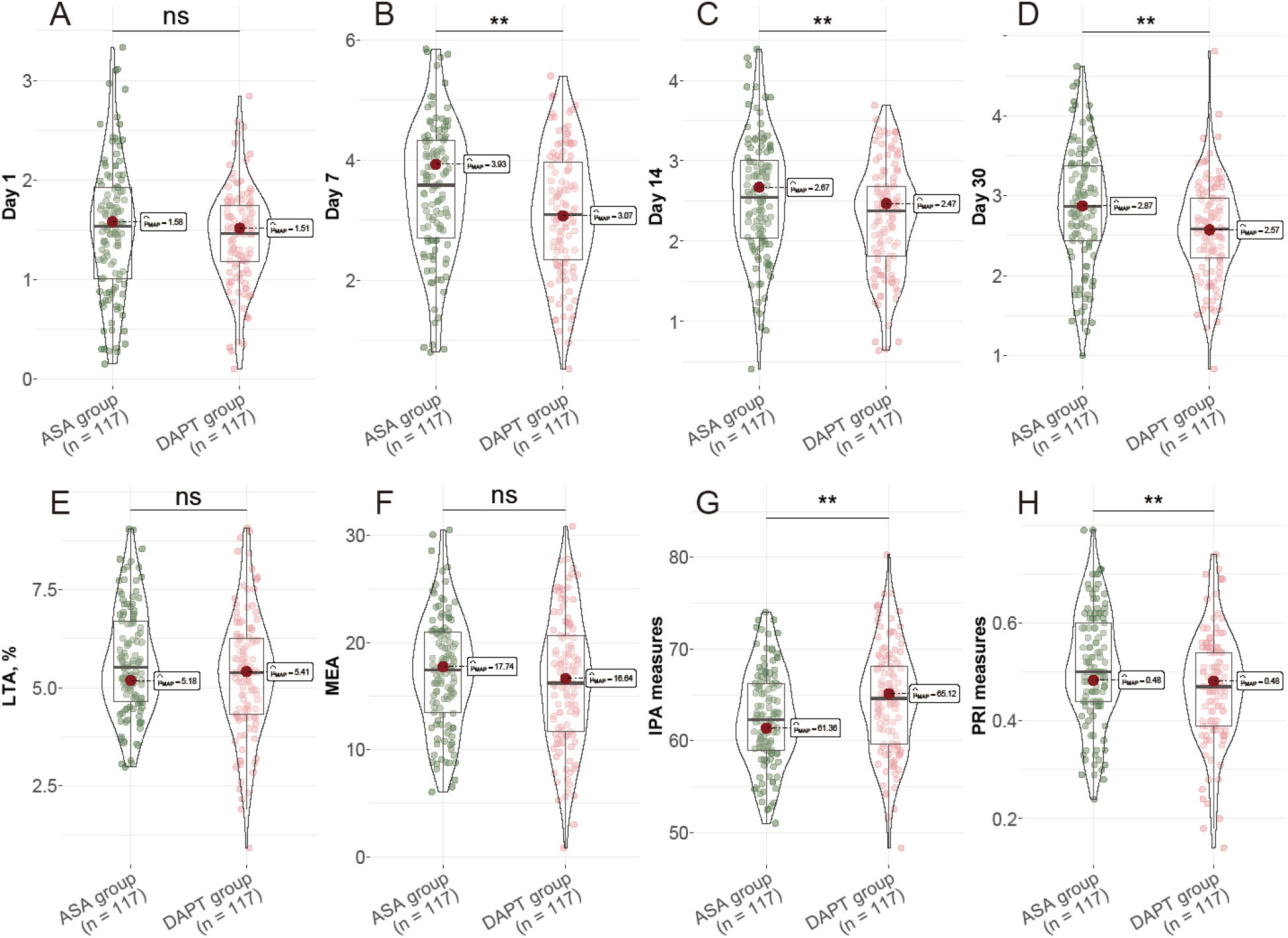
Postoperative Platelet Function Assessment. **(A)** Thromboxane B2 levels at day 1; **(B)** Thromboxane B2 levels at day 7; **(C)** Thromboxane B2 levels at day 14; **(D)** Thromboxane B2 levels at day 30; **(E)** LTA, %; **(F)** MEA, U; **(G)** IPA measures, %; **(H)** PRI measures, %. LTA: light transmission aggregometry; MEA: multiple electrode aggregometry; IPA: Inhibition of platelet aggregation; PRI: platelet reactivity index.

On day 1 following surgery, thromboxane B2 levels were similar between groups (ASA: 1.51 ± 0.67 pg/ml; DAPT: 1.43 ± 0.48 pg/ml; *P* = 0.332) (Figure 1). However, by day 7, thromboxane B2 levels were notably lower in the DAPT group (3.12 ± 1.05 pg/ml) compared to the ASA group (3.49 ± 1.11 pg/ml), with this trend continuing through days 14 and 30, where levels remained significantly reduced in the DAPT group (*P* = 0.009, *P* = 0.005, and *P* = 0.002, respectively). Although light transmission aggregometry (LTA) and multiple electrode aggregometry (MEA) values did not show significant differences between the groups (*P* = 0.082 and *P* = 0.246, respectively), the IPA was significantly enhanced in the DAPT group (64.35 ± 5.84%) compared to the ASA group (62.46 ± 5.23%, *P* = 0.010). Additionally, the PRI was significantly lower in the DAPT group at 0.47 ± 0.11 compared to the ASA group at 0.51 ± 0.12 (*P* = 0.007). These data indicate a stronger inhibition of platelet function and reduced platelet reactivity in the DAPT group, suggesting that the combination of clopidogrel with aspirin enhances antiplatelet effects, which may contribute to improved clinical outcomes in patients undergoing CABG.

The mean LVEF was slightly higher in the DAPT group (48.64 ± 9.03%) compared to the ASA group (47.36 ± 8.97%), but this difference was not statistically significant (*P* = 0.278) (Table 6). The distribution of LVEF categories also revealed no substantial differences between the groups, with 41.02% of ASA patients and 46.15% of DAPT patients falling in the ≥50% LVEF category. Similarly, the proportions of patients in the 40%–50% LVEF range were nearly identical, with 44.44% in the ASA group and 45.30% in the DAPT group. Fewer patients showed more pronounced reductions in LVEF, with 11.96% of ASA patients and 6.84% of DAPT patients in the 30%–40% range, and minimal representation in the <30% category for both groups (2.56% in ASA and 1.71% in DAPT). The degree of LVEF decrease did not differ significantly between the two groups (*P* = 0.532). Overall, these findings suggest that the addition of clopidogrel to aspirin does not adversely impact short-term postoperative cardiac function as measured by LVEF in patients undergoing CABG.

**Table 6:** Postoperative LVEF.

Parameters	ASA group (*n* = 117)	DAPT group (*n* = 117)	t/χ^2^	*P*
LVEF, %	47.36 ± 8.97	48.64 ± 9.03	1.088	0.278
LVEF decrease degree			2.199	0.532
≥50%	48 (41.02%)	54 (46.15%)		
40%–50%	52 (44.44%)	53 (45.30%)		
30%–40%	14 (11.96%)	8 (6.84%)		
<30%	3 (2.56%)	2 (1.71%)		

### 3.4 Postoperative prognosis and adverse event data

Incidences of major adverse cardiovascular and cerebrovascular events (MACCE), such as MI and cerebrovascular accidents (CVA), were slightly lower in the DAPT group, with 1.71% experiencing CVA compared to 5.98% in the ASA group, though this difference did not achieve statistical significance (*P* = 0.174) (Table 7). Re-hospitalization for any cause tended to be less frequent in the DAPT group (14.53%) compared to the ASA group (24.79%), yet this was not statistically significant (*P* = 1.000).

**Table 7:** Effects of aspirin alone vs. aspirin with clopidogrel in the first month post-surgery on adverse events.

Parameters	ASA group (*n* = 117)	DAPT group (*n* = 117)	χ^2^	*P*
Coma > 24 h	1 (0.85%)	0 (0.00%)	0.000	1.000
Renal failure	2 (1.71%)	1 (0.85%)	0.000	1.000
MACCE				
MI	4 (3.42%)	1 (0.85%)	0.817	0.366
CVA	7 (5.98%)	2 (1.71%)	1.849	0.174
Cardiac arrest	2 (1.71%)	1 (0.85%)	0.000	1.000
Late tamponade	3 (2.56%)	2 (1.71%)	0.000	1.000
Repeat revascularization	8 (6.84%)	3 (2.56%)	2.385	0.123
Minor bleeding				
Epistaxis	1 (0.85%)	8 (6.84%)	4.160	0.041
Other	2 (1.71%)	9 (7.69%)	4.674	0.031
Major bleeding				
Gastrointestinal	2 (1.71%)	3 (2.56%)	0.000	1.000
ICH	2 (1.71%)	2 (1.71%)	0.000	1.000
DVT	2 (1.71%)	2 (1.71%)	0.000	1.000
Ruptured AAA	1 (0.85%)	0 (0.00%)	None	0.048
Re-hospitalization any cause	29 (24.79%)	17 (14.53%)	3.896	1.000

MACCE, major adverse cardiovascular and cerebrovascular events; MI, myocardial infarction; CVA, cerebrovascular accident; ICH, intracerebral hemorrhage; DVT, deep vein thrombosis; AAA, abdominal aorta aneurysm.

Examining minor bleeding events, however, highlighted significant differences: epistaxis was notably more prevalent in the DAPT cohort, occurring in 6.84% of patients compared to 0.85% in the ASA group (*P* = 0.041). Similarly, other minor bleeding complications were more frequent in the DAPT group (7.69%) vs. the ASA group (1.71%) (*P* = 0.031). Meanwhile, major bleeding episodes such as gastrointestinal bleeding and intracerebral hemorrhage (ICH) remained comparable between groups, with no statistically significant difference. Additionally, a single case of ruptured abdominal aortive aneurysm (AAA) occurred in the ASA group with no corresponding incidents in the DAPT group (*P* = 0.048).

Overall, while DAPT exhibited a favorable profile in terms of reducing some MACCE, it was associated with increased minor bleeding events. These findings suggest a nuanced benefit-risk profile when considering DAPT in the immediate postoperative period following CABG, warranting careful patient selection and monitoring.

### 3.5 Regression analysis

Out of the total cohort of 281 patients, 57 experienced adverse events, resulting in an adverse event rate of 20.28% (Table 8). Treatment type emerged as a significant predictor of these events. Among patients treated with aspirin alone (ASA), 56.14% experienced adverse events, compared to 43.86% in the DAPT group, indicating a lower likelihood of adverse events with DAPT (*P* = 0.045 for ASA, *P* = 0.047 for DAPT). The odds ratio for experiencing an adverse event was 0.502 (95% CI: 0.314, 0.827), suggesting that patients on DAPT had approximately half the odds of adverse events compared to those on ASA alone. These results underline the potential benefit of combining clopidogrel with aspirin in reducing postoperative complications in the month following CABG, highlighting the clinical importance of tailored antiplatelet therapy strategies in this patient population.

**Table 8:** Regression analysis.

Parameters	Total	Adverse Event (Yes)	Adverse Event (No)	OR (95% CI)	*P*-value
Number of Patients	281	57	224	0.502 (0.314, 0.827)	/
Adverse Event Rate (%)	20.28%	/	/		/
Treatment Type—ASA (%)	125 (44.48%)	32 (56.14%)	93 (41.52%)		0.045
Treatment Type—DAPT (%)	156 (55.52%)	25 (43.86%)	131 (58.48%)		0.047

The logistic regression analysis presented in Table 9 provides a comprehensive evaluation of factors influencing adverse events in the first month following CABG. Treatment type emerged as a significant factor, with patients receiving DAPT showing a reduced likelihood of adverse events compared to those on aspirin alone (ASA), as indicated by an odds ratio (OR) of 0.452 (95% CI: 0.253, 0.816) and a *P*-value of 0.008. Age was also a significant predictor, with each additional year associated with a slight increase in odds (OR: 1.015, 95% CI: 0.991, 1.037; *P* = 0.003). Female patients demonstrated a notably lower risk of adverse events compared to males (OR: 0.503, 95% CI: 0.302, 0.841; *P* = 0.009).

**Table 9:** Logistic regression results for CABG prognosis adverse events.

Parameters	Odds Ratio (95% CI)	*P*-value
Treatment Type (DAPT vs. ASA)	0.452 (0.253, 0.816)	0.008
Age	1.015 (0.991, 1.037)	0.003
Gender (Female vs. Male)	0.503 (0.302, 0.841)	0.009
Baseline Health Status	0.951 (0.934, 0.976)	0.004
Diabetes	1.608 (1.054, 2.455)	0.028
Hypertension	1.355 (0.853, 2.125)	0.201
Previous MI	2.107 (1.304, 3.382)	0.002
Smoking Status	1.806 (1.105, 2.953)	0.019

Baseline health status had a protective effect, with better health correlating with reduced adverse event odds (OR: 0.951, 95% CI: 0.934, 0.976; *P* = 0.004). Conversely, diabetes significantly increased the risk of adverse events (OR: 1.608, 95% CI: 1.054, 2.455; *P* = 0.028), as did a history of previous MI, with the odds more than doubling (OR: 2.107, 95% CI: 1.304, 3.382; *P* = 0.002). Smoking status also heightened risk (OR: 1.806, 95% CI: 1.105, 2.953; *P* = 0.019), whereas hypertension did not achieve statistical significance in this model (OR: 1.355, 95% CI: 0.853, 2.125; *P* = 0.201). These findings highlight the importance of both treatment strategy and patient-specific clinical factors in influencing early postoperative outcomes following CABG.

## 4 Discussion

In this study, we assessed the impact of DAPT, comprising clopidogrel combined with aspirin, vs. aspirin alone on postoperative outcomes in patients undergoing CABG. One of the primary observations was the reduced incidence of graft occlusion in patients treated with DAPT compared to those on aspirin alone. The mechanism behind this improvement likely relates to the more effective IPA achieved with dual therapy (17). Clopidogrel, an ADP-receptor antagonist, works synergistically with aspirin to provide a more robust antiplatelet effect (18). By inhibiting different pathways in platelet activation, DAPT reduces thromboxane B2 levels over time and enhances IPA, as reflected in the significant decrease in PRI in the DAPT group. This comprehensive antiplatelet effect likely prevents occlusion, particularly in SVG, which were more prone to thrombosis due to their native pro-thrombotic endothelial environment compared to arterial grafts (19).

While DAPT was associated with a trend towards fewer major adverse cardiovascular and cerebrovascular events (MACCE) such as MI and cerebrovascular accidents (CVA), the statistical significance was not reached across the entire cohort. This may be attributed to the relatively short follow-up period of one month, as the enduring benefits of such enhanced antiplatelet regimens might be more apparent over longer durations (20, 21). However, our logistic regression analysis indicates a substantially lower odds ratio for adverse events in patients on DAPT compared to aspirin alone. This underscores an important interplay between antiplatelet therapy and overall cardiovascular event risk reduction, a relationship that has been well-documented in broader cardiovascular studies outside the perioperative CABG context.

Furthermore, the apparent protection conferred by DAPT against early graft failure, especially in the context of venous grafts, suggests an important consideration for personalized antiplatelet strategies in CABG patients (22, 23). Given the lack of significant difference in LVEF between treatment groups, it appears that the addition of clopidogrel does not compromise cardiac function, which supports its use from a hemodynamic standpoint (24).

The minor bleeding complications observed with DAPT—namely epistaxis and other minor bleeds—are consistent with the known risk profile of more intensive antiplatelet strategies (25). These findings align with concerns regarding bleeding risks inherent in any therapy that enhances anticoagulation, emphasizing the necessity of carefully weighing these risks, particularly in patients with a predisposed bleeding tendency or those who might be less tolerant of bleeding, such as the elderly or those with comorbid conditions like peptic ulcer disease (26, 27). Nonetheless, major bleeding events did not differ significantly between groups, which provides reassurance about the safety of DAPT in terms of catastrophic hemorrhage within this short-term postoperative window.

It was crucial to consider the clinical implications of predisposed factors that modulate the risk of adverse events. Our regression analysis identified age, gender, baseline health status, diabetes, and history of MI as significant predictors. For example, diabetes and previous MI, factors known to exacerbate platelet reactivity and endothelial dysfunction, heightened the risk of adverse events, and require careful monitoring and potentially more aggressive postoperative management (28, 29). Female gender appearing protective in our cohort adds to ongoing discussions in cardiothoracic circles regarding gender-specific responses to antiplatelet therapies (30). Understanding these nuanced interactions between patient characteristics and pharmacotherapy response remains an ongoing research priority (31).

The PSM ensured well-balanced baseline characteristics between treatment groups, minimizing confounding factors that could otherwise obscure true causal links (32, 33). Hence, the results likely reflect the genuine effects of the antiplatelet strategy rather than demographic or other clinical variables. This methodological robustness strengthens the argument for dual therapy in enhancing early postoperative CABG outcomes while highlighting the need for further subgroup analyses and extended follow-up studies to substantiate longer-term benefits and safety.

Another important consideration in optimizing the benefits of DAPT is the inter-individual variability in response to clopidogrel, which is strongly influenced by genetic polymorphisms—particularly those in the CYP2C19 enzyme. CYP2C19 loss-of-function alleles are associated with reduced formation of clopidogrel's active metabolite, resulting in diminished platelet inhibition and higher rates of adverse cardiovascular events after coronary interventions, including CABG. Previous study (34) have demonstrated that a genotype-guided strategy for antiplatelet selection can improve outcomes in patients with CYP2C19 polymorphisms by identifying individuals who may benefit from alternative P2Y12 inhibitors, such as prasugrel or ticagrelor. Although our study assessed platelet functional responses as an indicator of adequate antiplatelet effect, we did not implement CYP genotyping. Future research incorporating genotype-guided DAPT selection could further refine and individualize postoperative antiplatelet therapy in CABG patients. Besides, other approach can further optimize the effect of dual antiplatelet medication, such as Datta SS et al. (35, 36) reported thromboelastography Platelet Mapping as a useful preoperative tool to reduce transfusion requirement by determining timing of coronary artery bypass graft surgery in patients taking dual antiplatelet medication.

While this study provides valuable insights into the short-term impacts of DAPT vs. aspirin alone in patients undergoing CABG, several limitations must be acknowledged. The most significant limitation was the relatively short follow-up period of one month, which may not capture long-term outcomes and may overlook delayed adverse events or late benefits of the treatment regimens. Additionally, the study's sample size, although sufficient for detecting differences in immediate postoperative outcomes, may not be powered enough to determine rarer adverse events or to generalize the results to broader populations, especially considering variations in surgical techniques and postoperative management across different centers. Furthermore, the potential for residual confounding exists despite the use of PSM, as unmeasured variables could influence the outcomes. Besides, our reliance on clinical records may have inherent biases related to data completeness and accuracy. Future research should aim to address these limitations by incorporating longer follow-up periods, larger and more diverse populations, and multi-center involvement to validate and expand upon these findings. Another notable limitation is that our investigation focused exclusively on clopidogrel as the P2Y12 inhibitor in the DAPT regimen. Newer antiplatelet agents, such as prasugrel and ticagrelor, were not evaluated. These agents are known to provide more potent and consistent platelet inhibition than clopidogrel and have been associated with superior efficacy in acute coronary syndrome and percutaneous coronary intervention populations. However, they are also linked to a higher risk of postoperative bleeding—an important consideration in the immediate post-CABG period. As such, the findings from our study may not be fully extrapolated to DAPT regimens incorporating newer P2Y12 inhibitors, and further studies are warranted to explore their role and safety profile in the context of CABG.

## 5 Conclusion

In conclusion, our study contributes to the growing body of evidence supporting the efficacy of DAPT in CABG patients, particularly in preventing early saphenous vein graft occlusion and potentially reducing adverse cardiovascular events. However, it also reiterates the necessity of vigilant monitoring for minor bleeding events associated with such therapy. Tailoring antiplatelet strategies to individual patient profiles, considering risk factors like diabetes and previous MI, alongside continuous reassessment of bleeding risk, was essential for optimizing therapeutic outcomes. Future studies, ideally extending follow-up beyond the immediate postoperative period, were warranted to elucidate long-term effects and further refine antiplatelet protocols in CABG care pathways.

## Data Availability

The original contributions presented in the study are included in the article/Supplementary Material, further inquiries can be directed to the corresponding author.
